# COVID-19 und Lebererkrankungen

**DOI:** 10.1007/s11377-023-00680-9

**Published:** 2023-03-08

**Authors:** Stephan Schmid, Arne Kandulski, Martina Müller-Schilling

**Affiliations:** Klinik und Poliklinik für Innere Medizin 1, Gastroenterologie, Endokrinologie, Infektiologie und Rheumatologie, Universitätsklinikum Regensburg, Franz-Josef-Strauß-Allee 11, 93053 Regensburg, Deutschland

**Keywords:** Leber, Intensivmedizin, Sekundär sklerosierende Cholangitis, Akut-auf-chronisches Leberversagen, Angiotensin-converting-Enzym 2, Liver, Intensive care, Secondary sclerosing cholangitis, Acute-on-chronic liver failure, Angiotensin-converting enzyme 2

## Abstract

Bis zu 53 % der PatientInnen mit Coronavirus Disease 2019 (COVID-19) weisen eine hepatische Beteiligung auf. Durch die Expression der Hauptzielstruktur für „severe acute respiratory syndrome coronavirus type 2“ (SARS-CoV-2), des Angiotensin-converting-Enzym-2(ACE2)-Rezeptors, auch auf Cholangiozyten, sinusoidalen Endothelzellen und Hepatozyten kann es zu einer direkten Schädigung der Leber kommen. Ferner spielt eine indirekte (nicht durch Rezeptoren vermittelte) Schädigung der Leber im Rahmen von COVID-19 durch eine schwere systemische Inflammation mit Zytokinsturm, hepatischen Thrombosen und einer systemischen Hypoxie eine wichtige Rolle. Bei COVID-19 gelten Leberwerte als wichtige Prädiktoren für die Prognose der PatientInnen. Wichtig ist es hierbei Differenzialdiagnosen für die Leberwerterhöhung, wie andere Virusinfektionen, medikamentös-toxisch induzierte Leberschädigung sowie autoimmune, metabolische und andere Lebererkrankungen, abzuklären. Von hoher klinischer Relevanz für die Behandlung kritisch kranker PatientInnen auf der Intensivstation ist das Krankheitsbild der „secondary sclerosing cholangitis in critically ill patients“ (SSC-CIP). Hierfür sind unter anderem hochdosierte Katecholamine, eine Beatmung mit hohem positivem endexspiratorischem Druck (PEEP) und die extrakorporale Membranoxygenierung (ECMO) Risikofaktoren. Eine frühe Diagnose dieser Erkrankung und Behandlung mittels interventioneller endoskopischer retrograder Cholangiographie (ERC) ist hierbei von entscheidender Bedeutung. Auch sollte eine Lebertransplantation evaluiert werden. Bei einer COVID-19-Erkrankung treten Fälle mit SSC, sog. COVID-SSC, auf. Die COVID-SSC und die SSC-CIP sind im klinischen Phänotyp, Risikofaktoren, Prognose und transplantatfreien Überleben vergleichbar. PatientInnen mit vorbestehender Lebererkrankung haben kein erhöhtes Risiko für eine Infektion mit SARS-CoV‑2, erkranken jedoch schwerer an COVID-19 als PatientInnen ohne Lebervorerkrankungen. Bei PatientInnen mit einer vorbestehenden Leberzirrhose kann eine SARS-CoV-2-Infektion ein akut-auf-chronisches Leberversagen (ACLF) induzieren. Hierbei handelt es sich um ein Krankheitsbild mit einer sehr hohen Mortalität, das im Rahmen einer intensivmedizinischen Behandlung therapiert werden muss.

## Abstract (en)

In patients with coronavirus disease 2019 (COVID-19), hepatic involvement occurs in up to 53% of all cases. Via the primary target for severe acute respiratory syndrome coronavirus type 2 (SARS-CoV-2), the angiotensin-converting enzyme 2 (ACE2) receptor, expressed on cholangiocytes, sinusoidal endothelial cells, and hepatocytes, direct damage to the liver may occur. Furthermore, indirect (= not receptor-mediated) damage to the liver plays a crucial role in the context of COVID-19 due to severe systemic inflammation with cytokine storm, hepatic thrombosis, and systemic hypoxia. In COVID-19, liver enzymes are considered significant predictors of outcome. Thus, it is essential to rule out other causes of liver enzyme elevation, such as other viral infections, drug-induced liver injury, and metabolic, autoimmune and other liver diseases. Secondary sclerosing cholangitis in critically ill patients (SSC-CIP) is highly relevant in treating critically ill patients in the intensive care unit (ICU). Risk factors for SSC-CIP include high doses of catecholamines, high positive end-expiratory pressure (PEEP), and extracorporeal membrane oxygenation (ECMO) therapy. Early recognition of this disease and treatment by endoscopic retrograde cholangiography (ERC) is crucial. Furthermore, liver transplantation should be evaluated. Some patients with COVID-19 are diagnosed with SSC, which is termed COVID-19-associated SSC. COVID-19-associated SSC and SSC-CIP are comparable with regard to clinical phenotype, risk factors, prognosis, and graft-free survival. Patients with pre-existing liver disease are not at increased risk for infection with SARS-CoV‑2 but show more severe clinical courses of COVID-19 than patients without pre-existing liver disease. Patients with pre-existing liver cirrhosis may develop acute-on-chronic liver failure (ACLF) upon infection with SARS-CoV‑2. ACLF has a high mortality rate, which must be treated in the ICU.

Im Dezember 2019 wurde ein neuartiges Coronavirus, das später als „severe acute respiratory syndrome coronavirus type 2“ (SARS-CoV-2) bezeichnet wurde, in Wuhan (China) als Erreger schwerer Lungenentzündungen identifiziert [1, 2]. Die durch SARS-CoV‑2 hervorgerufene Infektionserkrankung wurde als Coronavirus Disease 2019 (COVID-19) definiert [3]. Während initial der Fokus auf respiratorischen Symptomen sowie auf Fieber, Myalgien, Müdigkeit und Geschmacksstörungen lag, rückten hepatische Manifestationen von COVID-19 erst später in den Fokus des Interesses, obwohl diese bei bis zu 52 % der PatientInnen beobachtet werden [2, 4]. Sie treten typischerweise zwischen 13 und 23 Tage nach Beginn der Erkrankung auf [5].

In diesem Artikel werden die direkten, d. h. die durch Angiotensin-converting-Enzym-2-Rezeptoren (ACE2) oder die transmembrane Serinprotease 2 (TMPRSS2) vermittelten, und die indirekten, d. h. die nicht durch Rezeptoren vermittelten Auswirkungen von COVID-19 auf die Leber dargestellt [6]. Hierbei wird neben den möglichen Pathomechanismen sowohl auf PatientInnen mit als auch ohne Lebervorerkrankungen eingegangen. Schwerpunkte liegen hierbei auf der gastroenterologischen, hepatologischen und infektiologischen Intensivmedizin sowie auf der interventionellen Endoskopie.

## Mechanismen der direkten hepatischen Schädigung

Als Hauptzielstruktur für SARS-CoV‑2 wurde der ACE2-Rezeptor identifiziert. Weiterhin spielt die TMPRSS2 der Zielzelle eine wichtige Rolle [7]. Die ACE2-Rezeptoren wurden nicht nur in der Lunge auf Typ-II-Pneumozyten, sondern insbesondere auch in der Leber auf Cholangiozyten, sinusoidalen Endothelzellen und Hepatozyten nachgewiesen [8–10]. Interessanterweise wurde gezeigt, dass bei PatientInnen mit Hepatitis-C-assoziierter Leberzirrhose gegenüber gesunden Vergleichspersonen eine bis zu 30fach erhöhte ACE2-Expression vorliegt [11]. Eine verstärkte ACE2- und TMPRSS2-Expression wurde auch bei PatientInnen mit nichtalkoholischer Steatohepatitis (NASH) und Adipositas beobachtet [12].

Der Infektionsweg von SARS-CoV‑2 in die Leber ist noch nicht vollständig bekannt

Der Infektionsweg von SARS-CoV‑2 in die Leber ist noch nicht vollständig bekannt, er könnte über die Blutbahn erfolgen [13]. Ebenso besteht ein Infektionsmöglichkeit durch direkte Aszension über die Gallenwege oder durch Translokation des Virus über die Pfortader nach Infektion von Enterozyten [14]. In Untersuchungen wurde bei 68 % der an COVID-19 verstorbenen PatientInnen SARS-CoV‑2 in der Leber detektiert. Hierbei wurde virales Nukleokapsidprotein in hepatischen Stamm‑/Progenitorzellen (HSPC), Cholangiozyten und Hepatozyten nachgewiesen [15, 16].

## Mechanismen der indirekten Leberschädigung

Neben der direkten Schädigung der Leberzellen durch SARS-CoV‑2 kann es im Rahmen von COVID-19 auch zu einer indirekten Schädigung der Leber kommen. Diese ist durch verschiedene Pathomechanismen bedingt [17–21]. Im Rahmen von COVID-19 kommt es zu einer schweren systemischen Inflammation und zu einer verstärkten Zytokinproduktion. So wurde beispielsweise gezeigt, dass Interleukin(IL)-6 mit dem Schweregrad von COVID-19 korreliert und dass ein erhöhtes IL‑6 mit einer Leberschädigung bei COVID-19 PatientInnen assoziiert ist [22].

Durch den „Zytokinsturm“ kann es zu Gefäßschäden mit Thrombosen kommen

Durch diesen „Zytokinsturm“ kann es wiederum zu Gefäßschäden kommen, die mit Endothelschäden, Hyperkoagulation und arteriellen sowie venösen Thrombosen einhergehen. So wurden hepatische Thrombosen bei bis zu 29 % der PatientInnen mit COVID-19 nachgewiesen. Weiterhin spielt eine mögliche systemische Hypoxie im Rahmen eines Acute Respiratory Distress Syndrome (ARDS) eine wichtige Rolle. Hieraus können eine ischämische Hepatitis oder eine sekundär sklerosierenden Cholangitis (SSC) resultieren [23]. Hierauf wird im Folgenden im Rahmen der Fokussierung auf sehr schwer an COVID-19 erkrankte PatientInnen auf der Intensivstation noch weiter eingegangen werden.

## Erhöhte Leberwerte bei COVID-19

Bis zu 84 % aller hospitalisierter PatientInnen weisen erhöhte Leberwerte auf. Hierbei ist die Glutamat-Pyruvat-Transaminase (GPT) in bis zu 39 % der wegen COVID-19 hospitalisierten PatientInnen erhöht, die Glutamat-Oxalacetat-Transaminase (GOT) in 63 % der Betroffenen [15, 19, 24, 25]. Erhöhte Leberwerte sind prognostische Marker, es besteht eine Assoziation zwischen Leberwerterhöhung und Verlauf der Erkrankung wie z. B. Schock, Aufnahme auf die Intensivstation, mechanische Beatmung und Überleben. Neben GOT und GPT sind eine Hypoalbuminämie sowie ein Anstieg der alkalischen Phosphatase (AP) Prädiktoren für eine erhöhte Mortalität [26].

Erhöhte Leberwerte sind prognostische Marker

Über die bereits beschriebenen direkten und indirekten Schädigung der Leber durch COVID-19 hinaus müssen stets auch Differenzialdiagnosen, wie andere Virusinfektionen (z. B. Hepatitisvirus A, B, C, D, E; Zytomegalievirus; Epstein-Barr-Virus; Herpes-simplex-Virus etc.), eine medikamentös induzierte Leberschädigung („drug induced liver injury“, DILI) sowie autoimmune, metabolische und weitere Lebererkrankungen, in Erwägung gezogen werden. Hier spielen neben mikrobiologischen und klinisch-chemischen Laboruntersuchungen Sonographie sowie Schnittbildgebungen eine wichtige Rolle [25]. Weiterhin sollte bei Verdacht auf eine kardiale Genese der Leberwerterhöhung eine Echokardiographie erfolgen. Eine bereits begonnene antivirale Therapie von Hepatitis B und C sollte nicht unterbrochen werden, falls die Patientin oder der Patient an COVID-19 erkrankt. Ebenso sollte eine antivirale Therapie bei neu diagnostizierter Hepatitis B durchgeführt werden, insbesondere falls eine immunsuppressive Therapie eingeleitet wird ([27, 28], siehe hierzu auch den Artikel von Markus Cornberg et al. in dieser Ausgabe von *Die Gastroenterologie*).

### Intensivpflichtige PatientInnen mit sekundär sklerosierender Cholangitis

Die COVID-19-PatientInnen, die aufgrund der Schwere ihrer Erkrankung auf eine Intensivstation aufgenommen werden, haben höhere Transaminasen als PatientInnen, die nicht auf einer Intensivstation hospitalisiert sind. Darüber hinaus ist bei kritisch kranken PatientInnen die Entwicklung einer sekundär sklerosierenden Cholangitis (SSC) eine wichtige Folgeerkrankung [29]. Bei der SSC handelt es sich um eine cholestatisch-inflammatorische Gallenwegserkrankung, die zu einem fibrotischen Umbau der intrahepatischen Gallengänge führt [24, 30]. Zu den typischen Laborbefunden einer SSC gehören erhöhte und steigende γ‑Glutamyltransferase(GGT)-Werte, die einen Spitzenwert von etwa dem 20- bis 50Fachen des oberen Grenzwerts erreichen, gefolgt von einer Erhöhung der AP und schließlich des Bilirubins. Die bei IntensivptientInnen auftretende Entität der SSC wird als „secondary sclerosing cholangitis in critically ill patients“, kurz SSC-CIP, bezeichnet.

Bei COVID-19 sollte auf eine hochdosierte Gabe von Ketamin möglichst verzichtet werden

Risikofaktoren hierfür sind unter anderem Hypotonie, hochdosierte Vasopressortherapie, Beatmung mit hohem positivem endexpiratorischem Druck (PEEP) und extrakorporale Membranoxygenierung (ECMO). Bei einer COVID-19-Erkrankung können schwere Verläufe mit SSC (COVID-SSC) auftreten. Die COVID-SSC und SSC-CIP sind im klinischen Phänotyp, Verlauf, Risikofaktoren, Prognose und transplantatfreien Überleben vergleichbar. Eine Übersicht über bereits veröffentlichte Studien zur COVID-SSC findet sich in Tab. 1 [24, 31]. Zudem wurde in einer Studie von Wendel-Garcia et al. ein möglicher Dosis-Wirkungs-Zusammenhang zwischen der Langzeitinfusion von Ketamin und dem Anstieg des Gesamtbilirubins sowie ein erhöhtes, ketaminassoziiertes Risiko einer COVID-SSC beschrieben [32]. Basierend auf diesen Daten sollte zur Langzeitanalgosedierung von mechanisch beatmeten COVID-19-PatientInnen soweit möglich auf eine hochdosierte Gabe von Ketamin verzichtet werden.

**Table 1:** 

Studie (Jahr)	Patientenzahl	Anwendung von Ketamin (%)	Vorbestehende Lebererkrankungen (%)	Lebertransplantation (*LTX*)	Verstorben im Follow-up (%)
Hunyady et al. [33]	24	n.v.	n.v.	13 % LTX erfolgt	35,4 (gesamte SSC-Kohorte)
Faruqui et al. [30]	12	n. v.	8	8 % LTX erfolgt und 33 % LTX gelistet oder evaluiert	33
Hartl et al. [34]	10	90	100	10 % LTX evaluiert	50
Mallet et al. [35]	5	100	40	20 % LTX gelistet	40
Bütikofer et al. [36]	4	100	0	25 % LTX gelistet	50
Meersseman et al. [37]	4	100	0	50 % LTX erfolgt	50
Ferreira et al. [38]	4	75	25	50 % LTX evaluiert	0

*n.v.* nicht verfügbar

**Figure 1:**
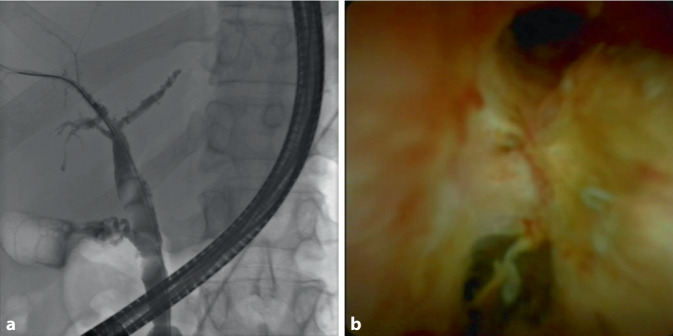

**Figure 2:**
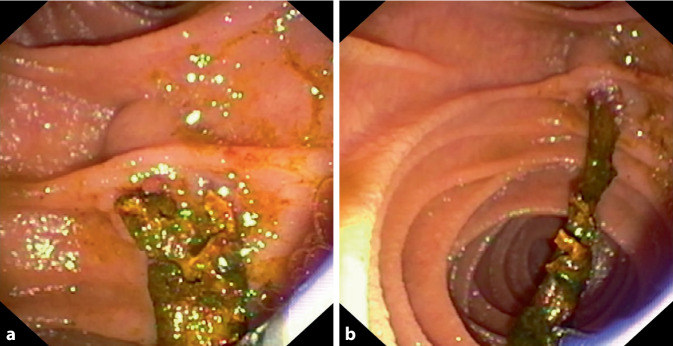

Es ist sehr wichtig, eine COVID-SSC frühzeitig zu erkennen und interventionell endoskopisch zu therapieren. Hierbei werden oft „casts“ und rarefizierte intrahepatische Gallengänge identifiziert (Abb. 1a). Cholangioskopisch zeigt sich häufig ein schwer destruiertes Gallengangsystem (Abb. 1b). Therapeutisch werden diese „casts“ extrahiert und die Stenosen dilatiert (Abb. 2; [23]). Insgesamt handelt es sich bei der COVID-SSC um eine sehr kritische Erkrankung mit einer sehr hohen Mortalität. Eine Lebertransplantation ist bei einer COVID-SSC zu evaluieren [39]. Wichtige Frühmarker für die Entwicklung einer SSC sind noch zu identifizieren, um mit geeigneten Beatmungs- und Sedierungskonzepten einen Progress womöglich zu verhindern [34, 40].

## COVID-19 bei vorbestehender Lebererkrankung

Aktuell vorliegende Studien zeigen, dass PatientInnen mit chronischen Lebererkrankungen keine erhöhte Anfälligkeit für eine Infektion mit SARS-CoV‑2 haben [6]. Eine große nordamerikanische Studie wies sogar nach, dass PatientInnen mit Leberzirrhose ein geringeres Risiko für eine SARS-CoV-2-Positivität haben [41]. Dies wurde unter anderem darauf zurückgeführt, dass sich PatientInnen mit Leberzirrhose häufig in einer engmaschigen ärztlichen Betreuung befinden und die entsprechenden Hygienemaßnahmen besser befolgen [6]. Für PatientInnen nach Lebertransplantation haben sich die Prävention und Therapieoptionen von COVID-19 durch Impfungen und antivirale Therapie zwischenzeitlich deutlich verbessert. Diesbezüglich wird auf den Artikel „Versorgung von Lebertransplantierten während der COVID-19-Pandemie – Update der S1 Leitlinie“ von Frau Dr. Leke Wiering und Herrn Prof. Dr. Frank Tacke in dieser Ausgabe von *Die Gastroenterologie* verwiesen.

### Leberzirrhose

#### Prognose

Im Fall einer Infektion mit SARS-CoV‑2 haben PatientInnen mit Leberzirrhose jedoch ein deutlich erhöhtes Risiko für einen ungünstigen Verlauf und eine erhöhte Mortalität. In einer großen Registerkohorte mit 729 PatientInnen aus 29 Ländern hatten PatientInnen mit einer Leberzirrhose nach einer SARS-CoV-2-Infektion eine Mortalität von 32 %.

Die Mortalität durch COVID-19 bei PatientInnen mit Leberzirrhose ist hoch

Dabei stieg die Mortalität von 8 % für PatientInnen mit Lebererkrankungen ohne Zirrhose über 19 % für PatientInnen mit einer Child-Pugh-A-Leberzirrhose und 35 % für PatientInnen mit einer Child-Pugh-B-Leberzirrhose auf 51 % für eine PatientInnen mit Child-Pugh-C-Leberzirrhose an [42]. Die Mortalität der PatientInnen mit einer Leberzirrhose stieg bei einer Krankheitsschwere, die eine Aufnahme auf die Intensivstation notwendig machte, noch weiter an, ebenso mit der Notwendigkeit von Nierenersatzverfahren und/oder mechanischer Beatmung. Die hohe Mortalität durch COVID-19 bei PatientInnen mit Leberzirrhose zeigte sich in der Folge in mehreren Studien weltweit [6, 42, 43].

#### Klinischer Verlauf und Langzeitprognose

Bei PatientInnen mit einer Leberzirrhose und COVID-19 kommt es in bis zu 46 % der Fälle zu einer Dekompensation der Leberzirrhose, die sich typischerweise durch neu aufgetretenen und sich verschlechternden Aszites und/oder eine hepatische Enzephalopathie zeigt. Von klinischer Relevanz ist, dass eine SARS-CoV-2-Infektion nicht selten (in 20 % bis 58 % der Fälle) auch ohne respiratorische Syndrome zur bereits beschriebenen Dekompensation der Leberzirrhose führen kann [42]. PatientInnen mit Leberzirrhose leiden zudem bei einer SARS-CoV-2-Infektion häufiger an gastrointestinalen Beschwerden als die Allgemeinbevölkerung. Dies ist unter anderem durch eine höhere Permeabilität des Darms bei PatientInnen mit Leberzirrhose zu begründen [44].

Die Heilungschancen eines ACLF nehmen mit zunehmendem Organversagen ab

Obwohl COVID-19 bei PatientInnen mit Leberzirrhose wie bereits dargestellt mit einem hohen unmittelbaren Sterberisiko verbunden ist, sind die Sterblichkeits- und Wiederaufnahmeraten nach 90 Tagen bei denjenigen, die den anfänglichen Insult überleben, mit jenen PatientInnen, die aufgrund einer Leberzirrhose, aber nicht wegen COVID-19 hospitalisiert sind, vergleichbar [50]. Daher scheint die SARS-CoV-2-Infektion nach der akuten Infektionsphase das Fortschreiten der Lebererkrankung über den natürlichen Verlauf der Zirrhose hinaus nicht zu beschleunigen [51].

### Indirekte Auswirkungen der SARS-CoV-2-Pandemie

Neben den direkten Auswirkungen der SARS-CoV-2-Infektion auf PatientInnen mit Leberzirrhose hatte die Pandemie aufgrund von Einschränkungen in der Gesundheitsversorgung auch weitreichende Auswirkungen auf die Versorgung der PatientInnen [52]. Dies führte zu einer verzögerten Vorstellung und Hospitalisierung schwer erkrankter PatientInnen mit fortgeschrittener Leberzirrhose und konsekutiv zu einer erhöhten Mortalität. Die Belastungen des Gesundheitssystems haben auch partiell zu Einschränkungen in der Surveillance des hepatozellulären Karzinoms (HCC) geführt, was zu einer verzögerten Vorstellung und einem fortgeschritteneren Tumorstadium bei Diagnosestellung im Vergleich zur Zeit vor der Pandemie führte [53].

### Akut-auf-chronisches Leberversagen

Zum akut-auf-chronischen Leberversagen (ACLF) kommt es bei 12–50 % der PatientInnen mit dekompensierter Leberzirrhose und COVID-19 [42, 43, 45, 46]. Das akut-auf-chronische Leberversagen (ACLF) ist durch eine akute Dekompensation einer chronischen Lebererkrankung definiert, die mit mindestens einem Organversagen einhergeht, und mit einer hohen Letalität assoziiert. Die in Europa gebräuchlichste Definition beruht auf einer Analyse der Daten aus der CANONIC-Studie Acute-on-Chronic Liver Failure in Cirrhosis der European Association for the Study of Chronic Liver Failure (EASL-CLIF). Die Diagnosekriterien für ein Organversagen zur Definition des ACLF basieren auf dem CLIF-Sepsis-related-organ-failure-assessment(SOFA)-Score. Hierbei handelt es sich um eine für PatientInnen mit chronischen Lebererkrankungen angepasste Version des SOFA-Scores [47].

Die Heilungschancen eines ACLF nehmen mit zunehmendem Organversagen ab. PatientInnen mit Child-Pugh-C-Zirrhose haben eine Überlebenschance von 21 %, wenn sie auf die Intensivstation aufgenommen werden. Diese sinkt auf 10 %, wenn es zum Lungenversagen kommt und eine mechanische Beatmung notwendig wird. Obwohl eine SARS-CoV-2-Infektion wie zuvor dargestellt eine Dekompensation einer vorbestehenden Leberzirrhose auslösen kann, ist die führende Todesursache (71 %) bei PatientInnen mit Leberzirrhose und COVID-19 überwiegend ein pulmonales Versagen gefolgt von durch die Leber bedingten Folgeerkrankungen und Organversagen (19 %; [6, 42]).

## Pathophysiologischer Zusammenhang zwischen Leber- und Lungenschädigung

Die Verschlechterung der Leberfunktion und die Lungenschädigung stehen bei einer SARS-CoV-2-Infektion in einem pathophysiologischen Zusammenhang. Hierbei spielen die zirrhoseassoziierte Immundysfunktion, die Koagulopathie, Aszites und die hepatische Enzephalopathie eine wichtige Rolle [21]. In Anbetracht der Tatsache, dass die Zusammensetzung der Darmmikrobiota die Immunreaktion des Wirts auf COVID-19 moduliert, ist es plausibel, dass Dysbiose und intestinale Permeabilität im Zusammenhang mit einer Leberzirrhose ebenfalls den Verlauf von COVID-19 aggravieren [48].

Bei COVID-19-PatientInnen mit Leberzirrhose kann es vermehrt zu Lungenembolien kommen

Interessanterweise spiegeln sich die Charakteristika einer dekompensierten Leberzirrhose, einschließlich der Aktivierung des Renin-Angiotensin-Aldosteron-Systems, der endothelialen Dysfunktion und der systemischen Entzündung, auch in der Pathophysiologie von COVID-19 wider [49]. Somit kann es zu einer Aggravierung kommen. Diese Veränderungen, sowohl durch die Leberzirrhose als auch durch COVID-19, führen auch zu einer gestörten Gerinnung. Dadurch kann es zu einer erhöhten Rate von Lungenembolien bei PatientInnen mit Leberzirrhose und COVID-19 im Vergleich zu PatientInnen mit COVID-19 ohne Leberzirrhose kommen [6, 49].

## Fazit für die Praxis

- Eine hepatische Beteiligung ist bei Coronavirus Disease 2019 (COVID-19) häufig und von hoher klinischer Relevanz.
- Im Rahmen von COVID-19 kann es zu einer direkten und indirekten Schädigung der Leber kommen.
- Leberwerte sind wichtige Prädiktoren der Prognose von PatientInnen mit COVID-19.
- Differenzialdiagnosen für Leberwerterhöhungen bei COVID-19, wie andere Virusinfektionen, medikamentös-induzierte Leberschädigung, sowie autoimmune, metabolische und weitere Lebererkrankungen, sollten stets abgeklärt werden.
- Bei kritisch kranken PatientInnen ist an die Entwicklung einer sekundär sklerosierenden Cholangitis (COVID-SSC) zu denken. Hier ist die frühzeitige Durchführung einer endoskopischen retrograden Cholangiopankreatikographie (ERCP) mit Extraktion von „casts“ eine wichtige Therapieoption. In schweren Fällen sollte eine Lebertransplantation erwogen werden.
- PatientInnen mit vorbestehenden Lebererkrankungen erkranken nicht häufiger, jedoch schwerer an COVID-19. Bei PatientInnen mit vorbestehender Lebererkrankung oder einer Leberzirrhose kann es durch COVID-19 zu einer Dekompensation mit Aszites und hepatischer Enzephalopathie bis hin zum akut-auf-chronischen Leberversagen (ALCF) kommen.
